# Imaging Pathways in Pediatric Thoracic Trauma: FAST-First Triage and Selective CT Escalation in Clinical Practice

**DOI:** 10.3390/diagnostics16060889

**Published:** 2026-03-17

**Authors:** Emil Radu Iacob, Emil Robert Stoicescu, Valentina Adriana Marcu, Roxana Stoicescu, Vlad Predescu, Narcis Flavius Tepeneu, Maria Corina Stanciulescu, Mihai Cristian Neagu, Adrian Georgescu, Calin Marius Popoiu

**Affiliations:** 1 Department of Pediatric Surgery and Orthopaedics, ‘Victor Babeș’ University of Medicine and Pharmacy, 300041 Timișoara, Romania; radueiacob@umft.ro (E.R.I.); tepeneu.narcis@umft.ro (N.F.T.); stanciulescu.maria@umft.ro (M.C.S.); mihai.neagu@umft.ro (M.C.N.); mcpopoiu@umft.ro (C.M.P.); 2 Department of Pediatric Surgery and Orthopaedics, ‘Louis Țurcanu’ Emergency Clinical Hospital for Children, 300011 Timișoara, Romania; marcuvalentina10@yahoo.ro; 3 Department of Radiology and Medical Imaging, ‘Victor Babeș’ University of Medicine and Pharmacy, 300041 Timișoara, Romania; stoicescu.emil@umft.ro; 4 Research Center for Medical Communication, ‘Victor Babeș’ University of Medicine and Pharmacy, Eftimie Murgu Square No. 2, 300041 Timișoara, Romania; 5 Department of Anatomy and Embriology, ‘Victor Babeș’ University of Medicine and Pharmacy, 300041 Timișoara, Romania; 6 Orthopaedics and Traumatology Department, Ponderas Academic Hospital, 014142 Bucharest, Romania; 7 Faculty of Physical Education and Sport, ‘Ovidius’ University of Constanța, 900470 Constanța, Romania; georgescu.adrian@univ-ovidius.ro

**Keywords:** pediatric thoracic trauma, imaging pathway, diagnostic sequencing, FAST, eFAST, chest radiography, computed tomography, pediatric imaging, thoracic trauma, pediatric population, chest trauma

## Abstract

**Background/Objectives**: Pediatric thoracic trauma requires prompt stabilization and timely imaging; however, actual sequencing and escalation triggers are infrequently delineated at the pathway level. The aim of this study was to analyze imaging pathways observed in routine clinical practice at our institution and to outline a preliminary escalation framework integrating injury mechanism, clinical severity, and initial ultrasound findings. **Methods**: A retrospective cohort study was conducted at the “Louis Țurcanu” Clinical Emergency Hospital for Children, Timișoara, Romania, including 66 children admitted with primary thoracic trauma between January 2022 and December 2024. Clinical trajectory markers (transfer-in, ICU admission, length of stay) and imaging utilization/sequencing (FAST, CXR, CT, MRI/CTA) were extracted. We divided injuries into two groups: bony (like fractures of the clavicle or scapula) and non-bony. CT escalation was characterized as a chest CT conducted upon admission. Fisher’s exact and Mann–Whitney U tests were used for comparative analyses. **Results**: FAST was done on all patients but was infrequently positive. Imaging followed heterogeneous but structured patterns, most commonly FAST with CXR, with or without CT. A large group of them had CT scans without first having any X-rays. CT escalation was associated with fracture-pattern injuries and higher-acuity trajectories (transfer-in and ICU admission), as well as prolonged hospital stays. Pathway-level assessment demonstrated that CT escalation effectively captured bony injury patterns, whereas FAST proficiently sorted ICU-level trajectories. **Conclusions**: Pediatric thoracic trauma imaging functioned as a selective escalation system: FAST served as a universal bedside entry step, and CT operated as an injury pattern- and acuity-linked severity gate. Making this escalation logic clear may help with standardization while still protecting against radiation.

## 1. Introduction

Trauma is one of the major causes of death in children worldwide, making up about 5–12% of all pediatric trauma admissions [1]. Thoracic trauma, while less common than head or abdominal injuries, is frequently clinically significant due to the potential involvement of vital organs, airway obstruction, and respiratory dysfunction [2]. The unique anatomical and physiological characteristics of children, including increased chest wall compliance and reduced protective musculature, predispose them to internal injury even in the absence of external signs [3,4]. Thoracic trauma can result from blunt mechanisms, predominantly motor vehicle collisions, falls, and sports-related injuries, as well as penetrating mechanisms, which are less common but linked to increased mortality [1,5].

An accurate and quick assessment of pediatric thoracic trauma is very important for making the right clinical decisions, from providing oxygen and ventilation support to doing procedures like placing a chest tube [6,7]. Misdiagnosis or delayed identification of pneumothorax, hemothorax, pulmonary contusions, or mediastinal air may result in respiratory failure, shock, or extended hospitalization [8]. Setting up a clear diagnostic pathway makes triage more efficient, makes better use of resources, and improves outcomes in situations where time is of the essence [6].

Initial management of pediatric thoracic trauma prioritizes rapid stabilization, airway protection, oxygenation, and circulatory support [9]. Primary assessment (Airway, Breathing, and Circulation—ABCs) identifies life-threatening injuries and guides immediate measures such as oxygen therapy, analgesia, or chest decompression [10]. A focused clinical examination and evaluation of the mechanism of injury help establish a provisional diagnosis [11]. Once the patient is stable, diagnostic imaging is necessary to confirm thoracic injuries and direct definitive management, making sure that imaging helps rather than slows down important treatments [3,9,12].

Imaging options for pediatric thoracic trauma include chest radiography (CXR), ultrasound, computed tomography (CT), and magnetic resonance imaging (MRI), each serving distinct roles in the diagnostic pathway [13]. Radiography enables swift screening, ultrasound facilitates bedside evaluation of pleural and pulmonary findings, CT offers high-resolution assessment in cases of suspected major trauma, and MRI is utilized for problem-solving in specific instances [14,15].

Despite widespread use of CXR, ultrasound, and CT in pediatric chest trauma, the sequencing of these modalities remains heterogeneous and is rarely quantified at the pathway level (i.e., which modality comes first, what triggers escalation, and how escalation relates to clinical trajectory) [16,17,18]. As a result, clinicians often rely on local practice rather than evidence-informed sequencing. In addition, objective factors such as equipment availability, institutional protocols, population characteristics, and legal or regulatory considerations may also contribute to this variability. This study addresses this gap by mapping modality sequencing and escalation triggers and translating them into a pragmatic imaging algorithm [8,14].

In pediatric trauma, ultrasound is widely used as a first-line imaging modality due to its rapid availability, lack of ionizing radiation, and bedside applicability. However, determining when escalation to CT or MRI is required remains challenging and often depends on clinical presentation, injury mechanism, and initial imaging findings.

Consequently, there is a need for structured imaging pathways that guide clinicians in selecting the most appropriate imaging modality while minimizing unnecessary radiation exposure.

The aim of this study was to analyze imaging pathways observed in routine clinical practice at our institution and to outline a preliminary escalation framework for pediatric thoracic trauma, intended to inform future prospective validation.

## 2. Materials and Methods

### 2.1. Study Design and Setting

A retrospective cohort study was conducted at the “Louis Țurcanu” Clinical Emergency Hospital for Children, Timișoara, Romania. Pediatric patients admitted with a primary diagnosis of thoracic trauma between January 2022 and December 2024 were identified through departmental records from Pediatric Orthopedics and Traumatology, Pediatric Surgery, and Plastic Surgery. After eligibility screening, 66 patients were included in the final analysis. The study was approved by the Ethics Committee of the ‘Louis Țurcanu’ Clinical Emergency Hospital for Children, Timișoara (approval no. 410/13.01.2025).

### 2.2. Eligibility Criteria

Patients were included based on clinical suspicion of thoracic trauma at presentation, while the specific injury diagnoses were subsequently established during imaging evaluation.

Inclusion criteria consisted of:

(1) Primary thoracic trauma diagnosis (e.g., thoracic contusion, pulmonary contusion, rib fractures, thoracic vertebral fractures, chest wall wounds);
(2) Hospitalization within the study interval;
(3) Clinical and imaging data must be available;
(4) Ultrasound examination (including Focused Assessment with Sonography for Trauma—FAST) was performed as the initial imaging investigation in all included patients; additional imaging modalities (radiography, CT, MRI, or CT angiography) were performed when clinically indicated;
(5) Informed consent must be documented and obtained from the patients’ legal guardians, as all participants were minors.

Patients were excluded if:

(1) Imaging examinations did not correspond to the anatomical region of trauma;
(2) Key clinical or imaging variables were missing from the medical records;
(3) The imaging protocol was incomplete;
(4) Documentation was insufficient for retrospective analysis.

### 2.3. Variables and Definitions

The following clinical variables were extracted: age, sex, BMI, transfer-in status (arrival to our institution from another hospital/service), ICU admission, length of stay (LOS), discharge against medical advice, and available laboratory parameters (arterial blood gas pH, when performed, creatine phosphokinase, and high-sensitivity D-dimer, when documented).

Ultrasound (including FAST when clinically indicated) was used as the initial imaging modality in most patients as part of the institutional trauma assessment protocol. FAST findings were recorded for all patients and evaluated as a binary variable (positive/negative) according to the documented ECO FAST results. When thoracic assessment was clinically indicated, the protocol was operationally extended to include pleural views (eFAST components) alongside the standard FAST windows, reflecting routine clinical practice at our institution. Additional imaging modalities such as CT or MRI were performed selectively depending on clinical findings, injury mechanism, and ultrasound results. MRI was not routinely performed after CT and was reserved for selected complex cases, and chest radiography was obtained when clinically indicated rather than systematically before CT. For injury pattern-driven analyses, injuries were categorized into:

- Bony thoracic injury pattern: fracture-pattern diagnoses (rib, sternal, and thoracic vertebral fractures; clavicle/scapula fractures were predefined as bony if present).
- Non-bony thoracic injury pattern: thoracic contusion, superficial thoracic injury/abrasion, and open thoracic wounds without fracture-pattern coding.

Thoracic vertebral fractures were included because they frequently occur in association with thoracic trauma mechanisms and may influence imaging decision pathways in pediatric trauma assessment.

CT escalation was defined as the performance of chest CT at any time during admission.

Imaging decisions were made within the institutional trauma assessment protocol by pediatric emergency physicians in collaboration with pediatric radiologists from our institution. Ultrasound examinations, including FAST assessments when indicated, were performed by pediatric radiologists with a minimum of three years of experience in pediatric radiology, while CT and MRI examinations were interpreted by board-certified radiologists experienced in pediatric imaging.

### 2.4. Statistical Analysis

Analyses were performed using Microsoft Excel, MedCalc^®^ Statistical Software version 23.0.9 (MedCalc Software Ltd., Ostend, Belgium), and Python v3.11 (pandas/SciPy). Continuous variables were tested for normality using the Shapiro–Wilk test and summarized as mean ± SD or median (IQR), as appropriate. Categorical variables were reported as *n* (%). Group comparisons used the Mann–Whitney U test for continuous variables and Fisher’s exact test for categorical variables. Odds ratios (ORs) with 95% confidence intervals (CIs) were computed using the Haldane–Anscombe correction when zero cells were present. Statistical significance was set at *p* < 0.05 (two-sided).

Because lesion-level imaging outcomes (e.g., “fracture detected on CT: yes/no”) were not encoded as binary report variables, modality performance was quantified as pathway-level/triage performance using clinically adjudicated injury pattern and trajectory endpoints as reference standards:

- Bony injury pattern as the reference condition for cross-sectional escalation; and
- ICU admission and transfer-in as trajectory endpoints for triage-signal assessment (FAST positivity).

Proportion CIs were computed with the Wilson method; LR CIs were computed on the log scale with continuity correction where applicable.

## 3. Results

### 3.1. Study Population and Baseline Characteristics

Sixty-six pediatric patients with thoracic trauma were analyzed. Mean age was 10.6 ± 3.9 years (median 11 [IQR 8–13], range 2–16), and 42 (63.6%) were male. BMI demonstrated a non-normal distribution (Shapiro–Wilk *p* < 0.001) and was summarized as 18.35 (15.40–23.90) kg/m^2^ (range 11.8–40.4). The overall median LOS was 2 (2–4) days (range 0–5). Transfer-in occurred in 18 (27.3%) patients, and 12 (18.2%) required ICU admission. Discharge against medical advice occurred in 3 (4.5%) cases. Baseline characteristics are provided in Table 1.

### 3.2. Thoracic Trauma Diagnostic Categories and Injury Patterns

Thoracic contusion was the most common diagnosis (30/66; 45.5%), followed by thoracic spine fractures (18/66; 27.3%), rib fractures (6/66; 9.1%), open thoracic wounds (6/66; 9.1%), sternal fracture (3/66; 4.5%), and superficial thoracic injury (3/66; 4.5%). Using the predefined injury pattern grouping, bony injuries accounted for 27/66 (40.9%) and non-bony injuries for 39/66 (59.1%) (Table 2).

The primary mechanism of injury was blunt trauma, with motor vehicle collisions and falls being the most common causes. Only a small number of patients needed surgery, while most were treated with less invasive methods. In most cases, neurological status was preserved at presentation, which is consistent with isolated thoracic injury patterns.

### 3.3. Imaging Utilization and Modality Selection

**Figure 1:**
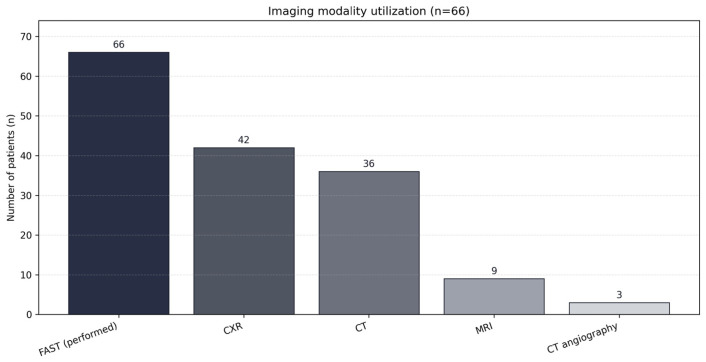
Imaging modality utilization frequencies.

FAST was performed in all patients (66/66; 100%) and was positive in 6/66 (9.1%). CXR was performed in 42/66 (63.6%), CT in 36/66 (54.5%), MRI in 9/66 (13.6%), and CT angiography in 3/66 (4.5%) (Table 3; Figure 1).

**Figure 2:**
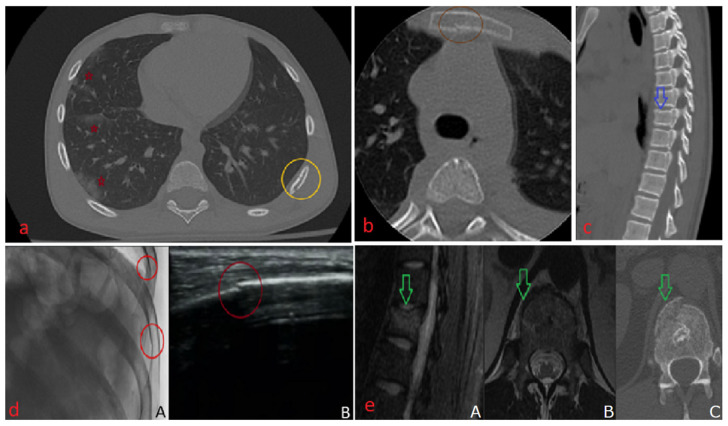
(**a**)—Chest CT, bone window, axial section shows two cortical disruptions of the left eighth rib arch (yellow circle), along with right pulmonary contusions—red stars; the patient had a left-sided flail chest. (**b**)—Spine CT, bone window, axial section reveals a unicortical disruption at the level of the sternum—orange circle. (**c**)—Spine CT, bone window, sagittal section reveals a compression fracture of the T8 vertebral body (blue arrow). (**d**)—(**A**). Rib series radiograph (inverted) shows cortical rib disruptions at the sites indicated by the red circles. (**B**). Ultrasound examination of the same patient demonstrates, within the red circle, a cortical disruption at the rib level. (**e**)—Green arrow shows (**A**). Sagittal MRI reconstruction; the STIR sequence shows a T12 compression fracture (~20% height loss), with a fracture line and STIR hyperintensity of the vertebral body consistent with bone marrow edema in an acute fracture. (**B**). The same fracture on axial T2-weighted MRI. (**C**). The fracture on CT, bone window.

The following Figure 2a–e shows representative imaging examples that show typical findings and the roles of different modalities in the observed workflow.

### 3.4. Diagnostic Sequencing Pathways in Clinical Practice

**Figure 3:**
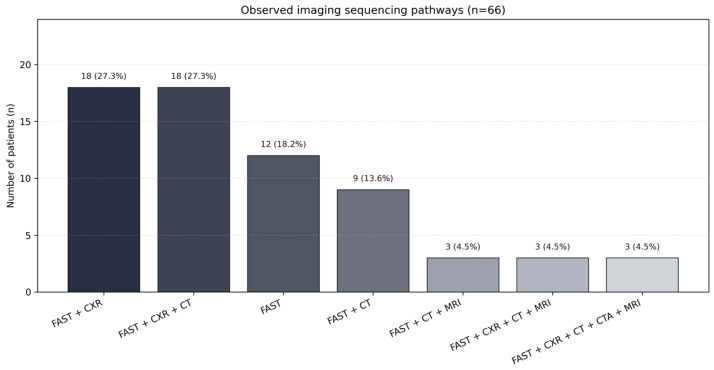
Observed imaging sequencing pathways.

Observed imaging trajectories demonstrated multiple pathway patterns. The most frequent were FAST + CXR + CT (18/66; 27.3%), FAST + CXR (18/66; 27.3%), FAST only (12/66; 18.2%), and FAST + CT (9/66; 13.6%). Less frequent multimodality pathways incorporated MRI and CTA (Table 3; Figure 3). Among all CT examinations, 12/36 (33.3%) were performed without prior CXR, indicating direct CT escalation in a substantial subset.

### 3.5. Resource Utilization and Clinical Outcomes

**Figure 4:**
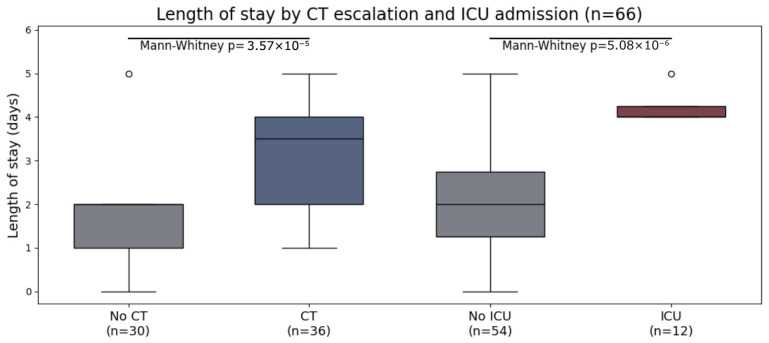
Length of stay stratified by CT utilization and ICU admission.

Median LOS differed significantly by escalation and acuity. Patients undergoing CT had longer LOS than those without CT (3.5 [2.0–4.0] vs. 2.0 [1.0–2.0] days; *p* < 0.001). ICU admissions also had prolonged LOS compared with non-ICU patients (4.0 [4.0–4.25] vs. 2.0 [1.0–3.0] days; *p* < 0.001). LOS was additionally higher in bony injury patterns versus non-bony (4.0 [3.0–4.0] vs. 2.0 [1.0–2.0] days; *p* < 0.001). LOS distributions are shown in Figure 4.

### 3.6. Associations Between Imaging Escalation and Phenotypic Markers

**Figure 5:**
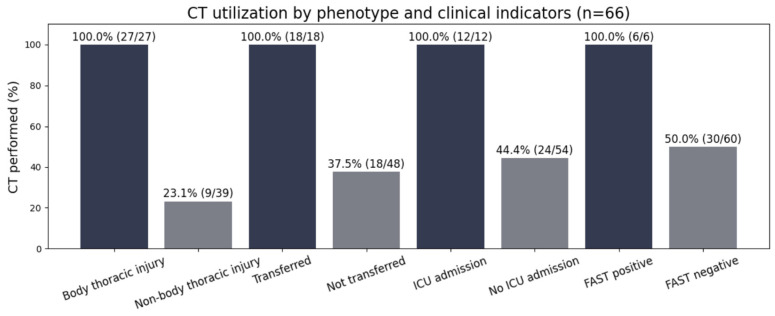
CT utilization by injury pattern and clinical indicators.

CT escalation clustered strongly around injury pattern and trajectory markers (Table 4). CT was performed in 100% of bony injury patterns (27/27) versus 23.1% of non-bony injury patterns (9/39) (*p* < 0.001), corresponding to OR 176.6 (95% CI 9.81–3177.86). All transfer-in patients underwent CT (18/18) compared with 18/48 non-transfer patients (*p* < 0.001; OR 61.0 [95% CI 3.47–1073.53]). CT was also universal among ICU admissions (12/12) versus 24/54 non-ICU admissions (*p* = 0.00027; OR 31.1 [95% CI 1.75–552.39]). FAST positivity was concentrated in ICU admissions (6/12 vs. 0/54, *p* < 0.001; OR 109.0 [95% CI 5.49–2165.95]). Figure 5 presents the distribution of CT utilization according to injury pattern and selected clinical indicators.

### 3.7. Laboratory Findings (Exploratory)

Creatine phosphokinase demonstrated substantial dispersion (median 111 (86–382), mean 569.8 ± 1244.9, range 61–5957). Arterial blood gas pH (*n* = 51) showed a stable mean of 7.364 ± 0.035 (range 7.279–7.422). High-sensitivity D-dimer (*n* = 15) ranged from 2312 to 18,760 (median 6252).

### 3.8. Synthesis Relevant to Imaging Algorithm Construction

**Figure 6:**
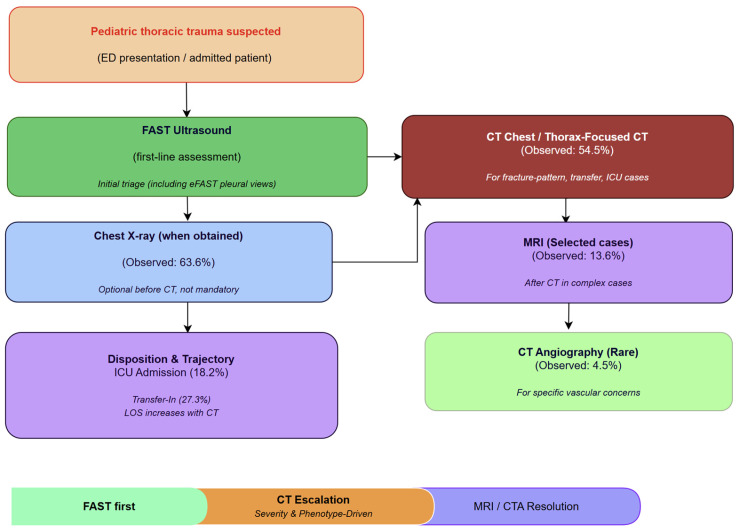
Proposed diagnostic imaging algorithm derived from observed patterns.

At the cohort level, imaging served as a two-stage diagnostic funnel: initial universal FAST screening succeeded by selective escalation to CT. Escalation was closely linked to fracture patterns and higher-acuity trajectories (transfer-in, ICU), which confirmed that CT is an operational severity gate with measurable resource implications, not just a standard middle step. Figure 6 shows a conceptual algorithm based on patterns that have been seen.

### 3.9. Diagnostic Performance (Pathway-Level and Triage Performance)

We were unable to figure out the formal lesion-level diagnostic accuracy (for example, “fracture detected on CT: yes/no”) because the report-level outcomes were not coded as binary variables. Instead, pathway-level performance was measured using (i) bony injury pattern as the reference condition for escalation and (ii) ICU admission/transfer-in as the trajectory endpoints for triage performance (FAST positivity).

CT escalation showed high sensitivity for bony injury pattern detection (100.0%, 95% CI 87.5–100.0) with specificity 76.9% (95% CI 61.7–87.4). FAST positivity showed strong rule-in properties for ICU admission: specificity 100.0% (95% CI 93.4–100.0), PPV 100.0% (95% CI 61.0–100.0), but limited sensitivity (50.0%, 95% CI 25.4–74.6), consistent with a triage-trigger signal. The diagnostic performance metrics can be found in the following table (Table 5).

### 3.10. Subgroup Signal and Diagnostic Implications (Sequencing + Clinical Impact)

CT escalation was injury pattern- and trajectory-dependent: 27/27 bony vs. 9/39 non-bony (*p* < 0.001), and 18/18 transfer-in vs. 18/48 non-transfer (*p* < 0.001). ICU admissions exhibited universal CT use (12/12) compared with 24/54 non-ICU (*p* = 0.00027). FAST positivity was concentrated in the ICU (6/12) versus none in the non-ICU (0/54, *p* < 0.001). LOS was significantly higher with CT use, ICU admission, and bony injury patterns (all *p* < 0.001) (Table 6).

## 4. Discussion

The initial management of pediatric thoracic trauma emphasizes physiological stabilization over definitive diagnosis, with imaging utilized solely to expedite or enhance management without postponing life-saving interventions. Pediatric trauma is markedly distinct from adult trauma owing to increased oxygen consumption, diminished cardiopulmonary reserve, and a pliable chest wall that may obscure clinically significant intrathoracic injury despite minimal external manifestations [8,19]. Accordingly, most pediatric trauma systems adopt an ABCDE approach that allows stabilization and triage to occur in parallel, while imaging is deployed as an escalation tool guided by physiological cues, suspected lesion pattern, and anticipated therapeutic consequences [6,20].

In this context, our cohort underwent a two-stage diagnostic funnel, with FAST serving as a universal, radiation-free entry point (incorporating pleural views when clinically warranted), while CT acted as a selective escalation. FAST abnormalities were infrequent but correlated with higher-acuity trajectories, indicating that FAST serves primarily as a rule-in triage trigger for early escalation and enhanced monitoring, rather than as a sensitive screening test for the exclusion of thoracic injury.

CT escalation was very selective and matched with higher-acuity trajectories and fracture-pattern injuries, such as transfer-in status and ICU admission. This finding suggests that CT is used less as a routine intermediate test and more as a practical “severity gate,” utilized when cross-sectional characterization is most likely to affect immediate decisions, procedural planning, and subsequent monitoring intensity [21,22]. It is important to note that escalation was linked to resource implications (for example, longer hospital stays in higher-acuity/CT-escalated pathways). This underscores that modality selection and sequencing are inherently operational decisions with downstream workflow implications.

From an implementation standpoint, the pathway can be integrated into the ED workflow as a concise, verifiable escalation logic: FAST-first by default, CXR for quick screening in stabilized low-risk cases and post-procedural verification, and CT escalation when injury pattern- or trajectory-triggers are present (suspected fracture-pattern injury, transfer-in status, persistent respiratory compromise despite stabilization, or a concerning bedside signal such as FAST positivity) [23]. This approach keeps clinical flexibility while making escalation logic clear, easy to reproduce, and easier to standardize across teams and shifts [24,25].

Recent guidance frames pediatric trauma imaging as a time-critical escalation process: bedside ultrasound and radiography are positioned to support rapid triage and early decision-making, while CT is recommended when the clinical picture suggests higher-risk injury or when cross-sectional definition is likely to change management. Our proposed pathway aligns with this escalation philosophy while introducing a workflow-level dimension by clarifying the sequencing logic—specifically, identifying which modalities are suitable and the timing and rationale for their activation in practical scenarios—thereby enhancing reproducibility and auditability among teams [26,27].

Our study not only describes the tests that were used but also how imaging was sequenced in routine clinical practice and what factors triggered escalation. Making these patterns into a clear, auditable path (FAST first, CXR when it adds value, CT when it changes decisions, MRI/CTA for targeted resolution) gives a practical template for standardization and validation across multiple centers [28,29].

CXR was still useful for quick screening and checking after a procedure, but a large number of people went straight to CT without having any prior radiography. This likely reflects an operational prioritization of time-to-comprehensive characterization in selected higher-risk scenarios, where CXR wouldn’t change immediate management much, while keeping CXR as a useful first-line tool in lower-risk, clinically stable presentations. In this situation, direct CT can speed up the process of finding and diagnosing lesions when a full characterization is needed right away, without taking away from the role of radiography in low-risk workflows.

Guideline discussions also recognize that imaging order is not strictly linear and may be modified according to acuity and anticipated subsequent requirements. In this context, the observed circumvention of CXR in favor of direct CT for specific higher-risk presentations can be regarded as a pragmatic streamlining of procedures rather than a rejection of radiography. The algorithm keeps CXR as a low-burden screening and post-procedural tool for stable cases, but it also knows that immediate cross-sectional imaging may be better when quick, detailed characterization is likely to affect disposition, monitoring intensity, or procedural planning [26,27].

Once the child was stable, MRI was only used a few times to help solve problems, mostly to clarify issues with the spine and soft tissue. This was in line with its known downstream role rather than its usefulness in acute triage. CT angiography was still rare and was only used as a targeted resolution tool for certain vascular issues. This showed that advanced modalities should still be used based on the patient’s needs rather than a set protocol in routine pediatric thoracic trauma.

In general, these patterns show that pediatric trauma imaging works more like a dynamic escalation system than a fixed ladder. It escalates when the injury pattern requires comprehensive characterization or when the clinical trajectory indicates severity. This framing makes sequencing and escalation triggers explicit, reproducible, and easier to audit across clinicians, shifts, and resource settings. Importantly, the present study analyzes imaging pathways observed in routine clinical practice at our institution and outlines a preliminary escalation framework that may serve as a basis for future prospective validation. Our retrospective single-center design, non-standardized imaging, and confounding-by-indication restrict causal inference; however, the identified escalation signals (fracture pattern, transfer/ICU trajectory, and FAST as an early triage signal) are clinically intuitive and likely applicable to similar pediatric emergency settings. In summary, FAST allows universal bedside triage, while CT determines severity and injury pattern. By making this escalation logic clear, we can move toward standardization. More research is needed to confirm performance and outcomes in larger multicenter cohorts.

## 5. Conclusions

In this study, imaging for pediatric thoracic trauma functioned as a selective escalation pathway rather than a definitive diagnostic hierarchy. FAST was a universal bedside triage step, while CT was used for injuries with a specific pattern of fractures and higher-acuity trajectories (transfer-in, ICU). This shows that CT is useful as a severity gate for full characterization. CT use was linked to longer hospital stays, which is what you would expect with more complicated injuries. CXR was still useful for quick screenings and checks after procedures in patients who were stable. MRI was only used to solve specific problems once the patient was stable. In general, these results support a FAST-first algorithm that uses injury pattern and acuity to decide when to use CT. This balances the need to protect patients from radiation with the clinical impact. The main point is operational: better outcomes come from better sequencing—triage first, escalate selectively, and only use advanced imaging when there is uncertainty that could change the decision.

## Figures and Tables

**Table 1:** Baseline characteristics.

Variable	Result
Age, years	10.6 ± 3.9 (median 11 [IQR 8–13]; range 2–16)
Sex, male	42 (63.6%)
BMI, kg/m^2^	18.35 (15.40–23.90); range 11.8–40.4
Transfer from another institution	18 (27.3%)
ICU admission	12 (18.2%)
Discharge against medical advice	3 (4.5%)
Length of stay, days	2 (2–4); range 0–5
Creatine phosphokinase (CPK), U/L	111 (86–382); mean 569.8 ± 1244.9; range 61–5957
Arterial pH (ASTRUP)	7.364 ± 0.035 (*n* = 51); range 7.279–7.422
D-dimer, ng/mL	6252 (*n* = 15); range 2312–18,760

**Table 2:** Thoracic trauma diagnoses and injury pattern stratification.

Diagnosis Category	*n* (%)	Phenotype
Thoracic contusion	30 (45.5%)	Non-bony
Thoracic spine fractures	18 (27.3%)	Bony
Rib fractures	6 (9.1%)	Bony
Open thoracic wound	6 (9.1%)	Non-bony
Sternal fracture	3 (4.5%)	Bony
Superficial thoracic injury	3 (4.5%)	Non-bony
Total bony	27 (40.9%)	—
Total non-bony	39 (59.1%)	—

**Table 3:** Imaging modality utilization and sequencing pathways.

Pathway	*n* (%)
FAST + CXR + CT	18 (27.3%)
FAST + CXR	18 (27.3%)
FAST only	12 (18.2%)
FAST + CT	9 (13.6%)
FAST + CXR + CT + MRI	3 (4.5%)
FAST + CT + MRI	3 (4.5%)
FAST + CXR + CT + CTA + MRI	3 (4.5%)
CT without prior CXR	12/36 CT (33.3%)

**Table 4:** Associations between imaging escalation and phenotypic/clinical indicators.

Comparison	*p*-Value (Fisher)	OR (95% CI)
CT in bony vs. non-bony injury pattern	<0.001	176.6 (9.81–3177.86)
CT in transfer-in vs. non-transfer	<0.001	61.0 (3.47–1073.53)
CT in ICU vs. non-ICU	0.00027	31.1 (1.75–552.39)
ICU in FAST+ vs. FAST−	<0.001	109.0 (5.49–2165.95)

**Table 5:** Diagnostic performance metrics (pathway-level/triage performance; mixed reference standard).

Test/Indicator	TP/FP/FN/TN	Sensitivity (95% CI)	Specificity (95% CI)	PPV (95% CI)	NPV (95% CI)	Accuracy (95% CI)	LR+ (95% CI)	LR− (95% CI)
CT escalation vs. bony injury pattern	27/9/0/30	100.0% (87.5–100.0)	76.9% (61.7–87.4)	75.0% (58.9–86.2)	100.0% (88.6–100.0)	86.4% (76.1–92.7)	4.14 (2.37–7.22)	0.02 (0.00–0.37)
CXR use vs. bony injury pattern	21/21/6/18	77.8% (59.2–89.4)	46.2% (31.6–61.4)	50.0% (35.5–64.5)	75.0% (55.1–88.0)	59.1% (47.0–70.1)	1.44 (1.01–2.06)	0.48 (0.22–1.05)
FAST positivity vs. ICU admission	6/0/6/54	50.0% (25.4–74.6)	100.0% (93.4–100.0)	100.0% (61.0–100.0)	90.0% (79.9–95.3)	90.9% (81.6–95.8)	55.0 (3.30–915.6)	0.50 (0.29–0.87)
FAST positivity vs. transfer-in	6/0/12/48	33.3% (16.3–56.3)	100.0% (92.6–100.0)	100.0% (61.0–100.0)	80.0% (68.2–88.2)	81.8% (70.9–89.3)	33.5 (1.98–566.6)	0.66 (0.48–0.92)

**Table 6:** Subgroup signal: imaging escalation, triage markers, and length of stay.

Analysis	Group 1	Group 2	Effect Size	*p*-Value
CT utilization by injury pattern	Bony: 27/27 (100.0%)	Non-bony: 9/39 (23.1%)	OR 176.6 (9.81–3177.86)	<0.001
CT utilization by transfer	Transfer-in: 18/18 (100.0%)	Non-transfer: 18/48 (37.5%)	OR 61.0 (3.47–1073.53)	<0.001
CT utilization by ICU	ICU: 12/12 (100.0%)	Non-ICU: 24/54 (44.4%)	OR 31.1 (1.75–552.39)	0.00027
FAST positivity by ICU	ICU: 6/12 (50.0%)	Non-ICU: 0/54 (0.0%)	OR 109.0 (5.49–2165.95)	<0.001
LOS by CT	3.5 (2.0–4.0)	2.0 (1.0–2.0)	—	<0.001
LOS by ICU	4.0 (4.0–4.25)	2.0 (1.0–3.0)	—	<0.001
LOS by injury pattern	4.0 (3.0–4.0)	2.0 (1.0–2.0)	—	<0.001

## Data Availability

The original contributions presented in this study are included in the article. Further inquiries can be directed to the corresponding authors.

## Abbreviations

The following abbreviations are used in this manuscript:

ABCs	Airway, Breathing, Circulation
ABCDE	Airway, Breathing, Circulation, Disability, Exposure
BMI	Body Mass Index
CI	Confidence Interval
CPK	Creatine Phosphokinase
CT	Computed tomography
CTA	Computed Tomography Angiography
CXR	Chest X-ray
eFAST	Extended Focused Assessment with Sonography for Trauma
FAST	Focused Assessment with Sonography for Trauma
ICU	Intensive Care Unit
IQR	Interquartile Range
LR	Likelihood Ratio
LOS	Length of stay
MRI	Magnetic resonance imaging
NPV	Negative Predictive Value
OR	Odds Ratio
PPV	Positive Predictive Value
SD	Standard Deviation
